# Effect of deep tissue laser therapy treatment on peripheral neuropathic pain in older adults with type 2 diabetes: a pilot randomized clinical trial

**DOI:** 10.1186/s12877-019-1237-5

**Published:** 2019-08-12

**Authors:** Prasun Chatterjee, Achal K. Srivastava, Deepa A. Kumar, Avinash Chakrawarty, Maroof A. Khan, Akash K. Ambashtha, Vijay Kumar, Luis De Taboada, Aparajit B. Dey

**Affiliations:** 1 Department of Geriatric Medicine, All India Institute of Medical Sciences, New Delhi, India; 2 Department of Neurology, All India Institute of Medical Sciences, New Delhi, India; 3 Department of Biostatistics, All India Institute of Medical Sciences, New Delhi, India; 4 Health World Hospital, Durgapur, West Bengal India; 5 LiteCure LLC, Carlsbad, CA USA

**Keywords:** Diabetic peripheral neuropathy, Older adults, Deep tissue laser therapy, Inflammatory markers

## Abstract

**Background:**

This study assessed the safety and efficacy of deep tissue laser therapy on the management of pain, functionality, systemic inflammation, and overall quality of life of older adults with painful diabetic peripheral neuropathy.

**Methods:**

The effects of deep tissue laser therapy (DTLT) were assessed in a randomized, double-masked, sham-controlled, interventional trial. Forty participants were randomized (1:1) to receive either DTLT or sham laser therapy (SLT). In addition to the standard-of-care treatment, participants received either DTLT or SLT twice weekly for 4 weeks and then once weekly for 8 weeks (a 12-week intervention period). The two treatments were identical, except that laser emission was disabled during SLT. Assessments for pain, functionality, serum levels of inflammatory biomarkers, and quality of life (QOL) were performed at baseline and after the 12-week intervention period. The results from the two treatments were compared using ANOVA in a pre-test-post-test design.

**Results:**

All participants randomized to the DTLT group and 85% (17 of 20) of participants randomized to the SLT group completed the trial. No significant differences in baseline characteristics between the groups were observed. After the 12-week intervention period, pain levels significantly decreased in both groups and were significantly lower in the DTLT group than in the SLT group. The Timed Up and Go test times (assessing functionality) were significantly improved in both groups and were 16% shorter in the DTLT group than in the SLT group. Serum levels of IL-6 decreased significantly in both groups. Additionally, serum levels of MCP-1 decreased significantly in the DTLT group but not in the SLT group. Patients’ quality of life improved significantly in the DTLT group but not in the SLT group.

**Conclusions:**

Deep tissue laser therapy significantly reduced pain and improved the quality of life of older patients with painful diabetic peripheral neuropathy.

**Trial registration:**

Clinical Trial Registry-India CTRI/2017/06/008739. [Registered on: 02/06/2017]. The trial was registered retrospectively.

## Background

India is considered the world’s capital of diabetes, with a projected diabetic population approaching the alarming mark of 70 million individuals by 2025 and 80 million by 2030 [1]. Individuals with diabetes are often unaware of the onset of diabetes due to the asymptomatic nature of this non-communicable disease (NCD) [2]. Diabetic peripheral neuropathy (DPN) is one of the most common complications of uncontrolled or poorly treated diabetes [3], and it is highly prevalent in older adults [4].

According to the members of an International Consensus Meeting, DPN is defined as “the presence of symptoms and/or signs of peripheral nerve dysfunction in people with diabetes after the exclusion of other causes” [5]. Various accepted theories related to DPN are changes in the blood vessels that supply the peripheral nerves, increased thickness of the basement membrane, loss of pericytes and endothelial hyperplasia [6], metabolic and autoimmune disorders accompanied by glial cell activation, changes in the expression of sodium and calcium channel, oxidative stress, and central pain mechanisms, such as increased thalamic vascularity and imbalance of the facilitatory/inhibitory descending pathways [7, 8]. The development of DPN is influenced by glucose tolerance, age, diabetes duration, alcohol consumption, and smoking [8, 9]. DPN has been associated with mobility limitation, impaired balance, falls and declines in functionality of older adults [10–14]. In addition, it interferes with sleep, daily activities, social interaction and mood, thereby compromising the overall quality of life [15, 16].

Recent research has indicated that proinflammatory cytokines, such as tumour necrosis factor-alpha (TNF-*α*) and interleukin-6 (IL-6) [17], and chemokines, such as monocyte chemoattractant protein-1 (MCP-1) and regulated on activation, normally T-cell expressed and secreted (RANTES), play critical roles in the pathogenesis of DPN [17].

DPN poses major treatment challenges to physicians in determining the appropriate pharmacological regimen, especially in older adults, due to inadequate symptom management, adverse drug interactions, or age-related changes in drug metabolism. Furthermore, the coexistence of multiple diseases (multimorbidity) with polypharmacy (more than 4 drugs) often complicates the choice of an effective treatment strategy [16]. To combat this situation, several nonpharmacological methods have emerged as alternate strategy for pain management in patients with DPN [18–23].

Studies have revealed that individual or multimodal exercise intervention improves balance, gait and mobility outcomes in patients with diabetic peripheral neuropathy [20, 21]. It has been demonstrated that intervention with aerobic exercise results in decreased pain interference as well as decreased general and physical fatigue [22]. However, physical activity for older adults with DPN should be performed with careful consideration of safety and modifications for optimal frequency, duration, and volume may be needed [23]. Deep tissue laser therapy (DTLT) is a nonpharmacological modality that uses non-ionizing wavelengths of laser energy, typically in the 600–1100 nm range, to affect cellular biochemistry in tissue repair and pain processes through photobiomodulation [19].

Given the large number of older adults with neuropathic pain and their underrepresentation in clinical trials on the nonpharmacological management of neuropathic pain, we conducted a randomized, double-masked, sham-controlled, interventional clinical trial involving 40 older adults (aged 60 years and above) with painful DPN (pDPN). This study was intended to determine whether the addition of DTLT to the standard-of-care pharmacological therapy could improve the multiple self-reported measures of pain, functional status, serum concentration of markers of inflammation, and patients’ overall quality of life.

## Methods

### Study ethics and registration

The study protocol was reviewed and approved by the ethics committee of the All India Institute of Medical Sciences, New Delhi, India (IEC-123/05.02.2016), and registered with India’s Clinical Trial Registry: CTRI/2017/06/008739. The trial was registered retrospectively on 02/06/2017. Informed consent was obtained from the participants prior to the commencement of the study.

### Study population

Study participants were recruited from individuals who were diagnosed with type 2 diabetes with pDPN and presented at the Department of Geriatric Medicine at AIIMS between April 2016 and September 2017. Participants’ pain was assessed using the neuropathic pain scale [24]. The scores were based on the responses to the questions about pain intensity. Positive neuropathic symptoms, such as numbness, tingling pain and increased pain due to touch, were noted, and neuropathy was confirmed by nerve conduction velocity (NCV) measurements [25]. Conventional NCV measurements were made using standard protocols, including testing of the bilateral peroneal motor nerves, sural sensory nerves, and sympathetic skin responses in the lower limbs [26]. Pain scores were taken, and NCV measurements were made by independent research staff who were blinded to all other parameters.

Participants were recruited irrespective of the duration of their pDPN; thus, the study included both newly diagnosed patients and patients with a long history of pDPN. Patients with type 1 diabetes, lower extremity open wounds, or psychotic, mood or neurological disorders that could interfere with the assessments were excluded. Patients with a life expectancy of less than 3 years and with any malignancy treated with chemotherapy or radiotherapy in the previous 2 years were also excluded from the study.

**Fig. 1:**
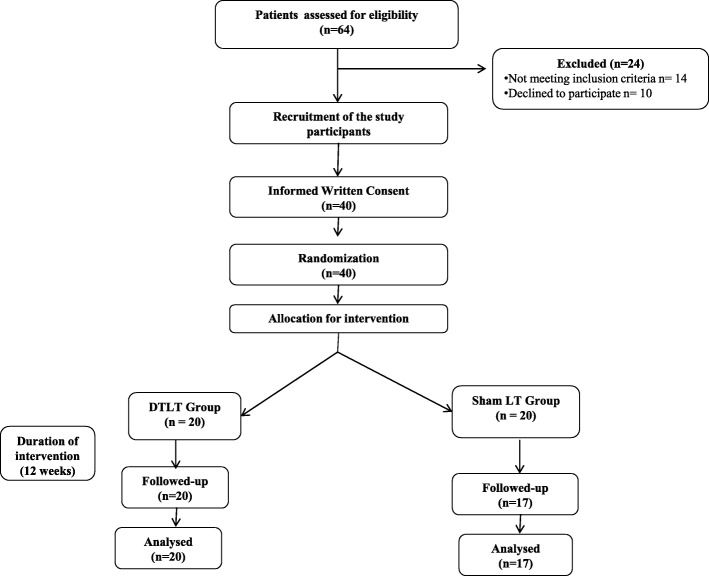
Flowchart showing the study design

Sixty-four patients with type 2 diabetes attending the Geriatric Medicine Department of AIIMS were screened for the inclusion/exclusion criteria. Forty patients meeting all the inclusion criteria and none of the exclusion criteria were selected for participation in the study (Fig. 1).

### Study design

The safety and efficacy of DTLT were assessed using a randomized, double-masked, sham-controlled, interventional trial. The enrolled participants were randomized (1:1) to either the DTLT group or the sham laser therapy (SLT) group (Fig. 1). Randomization was carried out with unique identity sequence generation and allocation concealment. A standard randomization procedure for allocation to each intervention stratum was generated using nQuery software version 2.0 (Statistical Solutions Ltd.). Allocation of the participants to the intervention group was performed using sequentially numbered, opaque, sealed envelopes. Group assignments for the participants were unknown to the participants, clinicians, and research staff.

During the study period, the participants were closely monitored for the occurrence of any possible adverse events such as erythema, hypersensitivity, or an unpleasant sensation of heating.

All personnel participating in the study were trained in the proper use of the laser device and the safe use of Class IV medical lasers prior to the commencement of the study. The laser device’s (LiteCure LLC, Newark, DE, USA) performance was verified to be in compliance with manufacturer specifications after installation at AIIMS and at the end of the 12-week intervention period.

### Treatment (intervention)

During the 12-week intervention period, study participants received either laser therapy (DTLT) or sham laser therapy (SLT) treatments twice weekly for 4 weeks and then once weekly for 8 weeks. Treatment consisted of the application of either DTLT or SLT to the plantar surfaces of the feet and to the lumbar spine, bilaterally from L4-S2. DTLT application to the lumbar spine was intended to stimulate the dorsal root ganglia and associated dermatomal patterns of the lower leg and foot. SLT treatment was identical to DTLT treatment, except in that in SLT, the laser emission was disabled.

Each plantar surface, an area of approximately 120–200 cm^2^, was treated using a blend of 980 nm and 810 nm laser light in an 80:20 optical power (W) ratio with an initial combined irradiance of 0.8 W/cm^2^ (2 W, continuous wave, 2.5 cm^2^ aperture). The treatment was applied using an in-contact, constant scanning motion technique (2.5 to 5 cm/second) for 3 min to deliver an initial dose of 360 J at 1.8–3.0 J/cm^2^ to each plantar surface. If no increased neuropathic foot pain was reported by the participant, the irradiance was increased by 0.4 W/cm^2^ (1 W, continuous wave) at their next session to a maximum of 4 W/cm^2^ (10 W, continuous wave). At the maximum applied irradiance, the dose delivered to each plantar surface was 1800 J at 9–15 J/cm^2^.

Each lumbar region, an area of 10 cm by 15 cm on each side of the spine, was treated using the same wavelength mix, optical power ratio, and treatment technique used to treat the plantar surfaces, with a fixed irradiance of 5.3 W/cm^2^ (8 W, continuous wave, 1.5 cm^2^ beam area). The treatment was applied for 4 min to deliver a dose of 1920 J at 13 J/cm^2^ during each treatment session.

### Outcome measures

Neuropathic pain, functional status, serum concentrations of inflammatory biomarkers and QOL were assessed at baseline and after the 12-week intervention period. Neuropathic pain was assessed using 3 different self-reported pain scales: the quadruple visual analogue pain scale (QVAS) [27], the neuropathic pain scale (NPS) [24], and the pain disability questionnaire (PDQ) [28]. The QVAS, a reliable and valid method for pain measurement, is based on four specific factors. The scores from the four factors are averaged and then multiplied by 10 to yield a score from zero to 100 [29]. The NPS is a comprehensive method for assessing pain in patients who have already been diagnosed with neuropathic pain. Scores were based on patient responses to questions about pain intensity [24]. The PDQ is a comprehensive psychometric evaluation of functional status. The primary focus of the PDQ is on disability and function. It is composed of two factors: a functional status component, with a maximum score of 90, and a psychosocial component, with a maximum score of 60. The PDQ yielded a total functional disability score ranging from zero to 150 [29].

Quality of life (QOL) was assessed via Short Form 36 (SF-36) [30], which included domains such as general health, functionality, physical health, bodily pain, interference with physical and emotional problems, and pain while carrying out social activities [30]. Functional status was assessed by a physiotherapist using a Timed Up and Go (TUG) test [31]. The TUG test is one of the quickest and best tools to assess the functionality and lower-limb muscle strength of older adults and to predict long-term morbidity [32]. For the TUG test, participants were instructed to rise from an armless chair, walk for 3 m in their usual manner and at a normal pace, turn around, walk back, and sit on the chair again. Participants performed the test with or without footwear or gait aids. The time it took the participant to perform the tasks was noted using a stopwatch. Timing was started as the word “Go” was uttered and stopped when the participant was again seated correctly, reclined in a chair [31].

Five millilitres of blood was collected from each participant at baseline and after the 12-week intervention period. The serum was prepared by centrifuging the blood immediately after collection in a Z383K centrifuge (Hermle Labortechnik, Wehingen, Germany) at 2500 RPM for 15 min. The serum was stored at − 80 °C in an ultralow Kaltis freezer prior to testing.

The serum samples were tested by an enzyme-linked immunosorbent assay (ELISA) [33] for the levels of the inflammatory biomarkers IL-6, TNF-α, MCP-1, and RANTES using commercially available Quantikine® ELISA kits (R&D Systems, Inc., a Bio-Techne brand, Minneapolis, MN, USA). Concentrations for each sample were calculated per the manufacturer’s instructions.

### Statistical analysis

To determine whether there were any differences in the baseline demographic profiles of the study groups, we compared each demographic characteristic using either a t-test or chi-square test, as appropriate. Differences were considered significant at *p-values <  0 .05*. To determine whether the treatment was effective, we compared the study’s outcomes (Tables 2, 3, 4 and 5) in a randomized pre-test-post-test design, using a two-factor ANOVA, followed by individual t-test comparisons of outcome metric means within groups. A significant difference in the pre-test-post-test means of an outcome metric within a group was taken to indicate that the group’s intervention – laser treatment or sham treatment – affected the outcome. A significant ANOVA interaction in the pre-test-post-test change between groups, i.e., change favouring DTLT, was taken as evidence of treatment efficacy. For measures for which the interaction was significant, unpaired t-tests were used to further explore the differences in change between the groups. Differences were considered significant at *p-values <  0.05*.

## Results

### Compliance with the protocol

All participants randomized to the DTLT group and 85% of participants (17 of 20) randomized to the SLT group completed the trial. No participant reported feeling heating or increased pain during treatment, thereby allowing all participants to comply with the treatment protocol. Participants in the DTLT group were able to receive the predetermined maximum plantar surface irradiance while maintaining the double-masked trial design. All participants in the DTLT group received the same total dose of irradiation during the 12-week intervention period.

### Adverse events

No adverse events were reported during the study period.

### Pre-test demographics

Forty participants (17 males and 23 females) were enrolled in the study. Table 1 shows the pre-test (baseline) demographic profiles of each group.

**Table 1:** Baseline demographic profiles

Characteristic	DTLT (*n* = 20)	SLT (*n* = 20)	*p*-value
Age (years +/− SD)	65.05 ± 5.54	64.1 ± 4.09	*0.541*
Sex			
Male	50%	35%	*0.52*
Female	50%	65%	
Education			
Illiterate	25%	55%	*0.100*
Primary	40%	15%	
Secondary and above	35%	30%	
Duration of diabetes (years +/− SD)	10.8 ± 8.12	11.44 ± 9.26	*0.489*
Fasting blood sugar (mg/dl +/− SD)	154.7895 ± 44.91	187 ± 68.73	*0.119*
Postprandial blood sugar (mg/dl +/− SD)	238.11 ± 55.25	248.28 ± 80.75	*0.662*

### Baseline demographic comparisons

The analysis showed that there were no significant differences in the baseline demographic characteristics of the groups, confirming the random assignment of study participants to the study groups (Table 1).

### Age

The average age of the participants was 65.05 years in the DTLT group and 64.1 years in the SLT group. The difference in mean age between the groups was not significant (*p = 0.541*) (Table 1).

### Sex distribution

Ten men and 10 women and 7 men and 13 women were randomized to the DTLT and SLT groups, respectively. While there was an equal distribution of male and female participants in the DTLT group, the percentage of women was higher in the SLT group. However, the difference in sex distribution between the groups was not significant (*p = 0.52*) (Table 1).

### Education level distribution

Five participants in the DTLT group and 11 participants in the SLT group were illiterate. Eight participants in the DTLT group and 3 participants in the SLT group had a primary education level. Seven participants in the DTLT group and 6 participants in the SLT group had secondary or higher education levels. While illiteracy was found to be higher for patients in the SLT group than in the DTLT group, those with primary and secondary and higher education levels were more frequently found in the DTLT group than in the SLT group. However, the difference in education levels between the groups was not significant (*p = 0.100*) (Table 1).

### Duration of diabetes

The average duration of diabetes was 10.8 years for participants in the DTLT group and 11.4 years for participants in the SLT group. The difference in duration of diabetes between the groups was not significant (*p = 0.489*) (Table 1).

### Fasting and postprandial blood sugar level

The average fasting blood sugar level was 155 mg/dl for participants in the DTLT group and 187 mg/dl for participants in the SLT group. The difference was not significant (*p = 0.119*) (Table 1). The average postprandial blood sugar level was 238 mg/dl for participants in the DTLT group and 248 mg/dl for participants in the SLT group. The difference in blood sugar levels between groups was not significant (*p = 0.662*) (Table 1).

### Outcome metrics

This study measured quality of life, pain, functionality, and systemic inflammation to assess intervention effects. Tables 2, 3, 4 and 5 show the observed pre- and post-intervention means for all outcomes by study group.

**Table 2:** Quality of life

Test	Group	Pre-test		Post-test		Percent Change
		Mean	+/− SD	Mean	+/− SD	
SF-36	DTLT	52.68	17.82	78.30	13.11	+ 49%
	SLT	61.47	15.35	66.01	14.69	+ 7%

*SF-36* 36-Item Short Form Survey

**Table 3:** Pain

Test	Group	Pre-test		Post-test		Percent Change
		Mean	+/− SD	Mean	+/− SD	
PDQ	DTLT	77.80	30.87	31.01	21.43	−60%
	SLT	62.94	22.03	45.29	18.16	−28%
QVAS	DTLT	69.66	19.46	31.82	19.60	−54%
	SLT	56.86	15.07	39.09	17.68	−28%
NPS	DTLT	0.75	0.81	−0.61	0.72	− 183%
	SLT	0.30	0.78	− 0.14	0.88	−150%

*PDQ* pain disability questionnaire, *QVAS* quadruple visual analogue, *NPS* numeric pain scale

**Table 4:** Functionality

Test	Group	Pre-test		Post-test		Percent Change
		Mean	+/− SD	Mean	+/− SD	
TUG	DTLT	17.85	7.04	13.00	3.24	−27%
	SLT	14.23	4.13	12.15	3.16	−11%

*TUG* Timed Up and Go

**Table 5:** Systemic inflammation

Test	Group	Pre-test		Post-test		Percent Change	*p*-value
		Mean	+/− SD	Mean	+/− SD		
IL-6	DTLT	0.84	0.54	0.67	0.27	−20%	= 0.038
	SLT	0.71	0.11	0.65	0.13	−8%	= 0.037
MCP-1	DTLT	4.91	1.59	4.10	1.25	−7%	= 0.037
	SLT	4.34	1.22	4.15	0.92	−4%	= 0.581
RANTES	DTLT	631.50	198.37	570.93	184.54	−10%	= 0.364
	SLT	651.80	205.56	606.56	164.92	−7%	= 0.482
TNF-*α*	DTLT	0.44	0.06	0.49	0.11	+ 11%	= 0.099
	SLT	0.44	0.07	0.47	0.12	+ 7%	= 0.285

*IL-6* interleukin-6, *MCP-1* monocyte chemoattractant protein-1, *RANTES* regulated on activation, normally T-cell expressed and secreted, *TNF-α* tumour necrosis factor alpha

Pre-test-post-test changes in mean SF-36 scores (Table 2) indicated that treatment (DTLT) significantly improved the QOL of participants in the study (*p <  0.001*), while the sham (SLT) treatment did not (*p = 0.194*), and that, when compared to the sham, the DTLT treatment significantly improved QOL (*p <  0.001*) (Table 6).

**Table 6:** Statistical analysis

Outcome	Test	Group	ANOVA Pre-test-Post-test Main Effect	ANOVA Interaction *p*-value	Pre-test vs Post-test
Quality of Life	SF-36	DTLT	< .001	< .001 ^a^	< 0.001
		SLT			= 0.194
Pain	PDQ	DTLT	< .05	< .01 ^a^	< 0.001
		SLT			< 0.01
	QVAS	DTLT	< .001	< .01 ^a^	< 0.001
		SLT			= 0.003
	NPS	DTLT	< .001	< .01 ^a^	< 0.001
		SLT			= 0.019
Functionality	TUG	DTLT	< .001	> .05 ^b^	
		SLT			
Systemic Inflammation	IL-6	DTLT	< .05	> .05 ^b^	
		SLT			
	MCP-1	DTLT	> .05	> .05 ^b^	
		SLT			
	RANTES	DTLT	> .05	> .05 ^b^	
		SLT			
	TNF-α	DTLT	> .05	> .05 ^b^	
		SLT			

^a^ For these comparisons, the ANOVA interaction was significant, and unpaired t-tests were used to further explore the differences in change between the groups. The t-tests showed that there were significant differences in the pre-test-post-test mean change of the SF-36, PDQ, QVAS and NPS metrics; in each case, the change was greatest for the DTLT group – taken to be indicative of treatment effect

^b^ For these comparisons, the ANOVA interaction was not significant. The pre-test vs post-test comparison in the ANOVA suggested that the mean values of the TUG test and IL-6 serum concentration decreased from pre-test to post-test and that there were no significant pre-test-post-test changes in the mean values of MCP-1, RANTES, and TNF-α

The ANOVA comparison of pre-test-post-test changes indicated a significant reduction in all measures of pain in both groups (Table 6). Specifically, the pre-test-post-test reduction in the mean PDQ score was significant in both groups (*p <  0 .001* for DTLT*; p <  0.01* for SLT), as were the reductions in QVAS (*p <  0.001* for DTLT*; p = 0.003* for SLT) and NPS (*p <  0.001* for DTLT*; p = 0.019* for SLT) scores (Table 6). The ANOVA interaction indicated that the reductions in PDQ, QVAS, and NPS pain scores were significantly different between groups (*p < 0.01 for all scales*), and they were greater in the treated group than in the sham group by 32, 26, and 33%, respectively (Table 3).

The ANOVA test of pre-test-post-test change in the mean TUG scores indicated that functionality was significantly improved in both groups compared to functionality at baseline (*p < 0 .001*) (Table 6)*.* The improvement in the mean TUG score was 16% greater in the treated group when compared to the sham group (Table 4). However, the ANOVA interaction indicated that the difference was not significant (*p > 0 .05*) (Table 6).

Analysis of the changes in mean serum levels of the systemic inflammation markers showed mixed results (Table 5). ANOVA of the pre-test-post-test serum levels of IL-6 indicated that systemic inflammation was significantly reduced from baseline in both groups (*p < 0 .05*). The mean serum levels of MCP-1 were significantly lower at post-test than at pre-test in the DTLT group (*p = 0 .037*) but not in the SLT group (*p* = 0.581) (Table 5). The pre-test-post-test analysis of serum levels of RANTES and TNF-α indicated no significant changes in systemic inflammation (*p > 0.05 for both metrics*). The changes in mean IL-6, MCP1, RANTES, and TNF-α scores were 12, 3, 3, and 4% greater, respectively, in the treated group than in the sham group (Table 5). However, the ANOVA interaction indicated that the differences were not significant (*p > 0 .05)* (Table 6).

The results of the analyses, as displayed in Table 6, suggest that the laser treatment was more effective than the sham at improving quality of life and reducing pain (all scales) and that there were no significant differences between groups in improvement of functionality or decrease in systemic inflammation.

## Discussion

The present study revealed a promising finding that deep tissue laser therapy (DTLT) administered for a period of 12 weeks was effective in managing pain in older adults with DPN. Although pain reduction was observed even in the SLT control group, the reduction in pain was significantly greater in the DTLT group than in the placebo group for all the evaluated pain scales. Thus, this study clearly indicates that DTLT is an effective method for managing DPN-associated pain in older adults. Pain reduction in the placebo group can be explained as an outcome of the pharmacological therapy received by the study participants. A study conducted by Cg et al. [19] from India observed that laser therapy was effective in pain management of DPN. However, the study analysed only pre-post findings within the group, as there was no control group [19]. Such uncontrolled trials pose limitations in interpreting the results, as the inclusion of a control group is critical to understanding the consequences of a specific intervention [26]. Our study mirrors the observation by Zinman et al. [26], which showed a decreased weekly pain score, as assessed by the visual analogue scale, following sham laser therapy for 2 weeks, with a further reduction in the pain score in the laser therapy group compared to controls after the 6 weeks of the study. A similar study concluded that patients who received laser therapy showed improved neuropathy outcomes compared to those of the sham or control group [34]. While these previous studies were conducted on participants from various age groups, our study was exclusively composed of older adults, aged 60 years and above, to observe the impact of laser therapy on their functionality, which is one of the vital determinants of active and healthy ageing.

Nonpharmacological means of intervention are a much-needed alternative in older adults, considering that most of the first-line drugs, such as tricyclic antidepressants (TCAs, such as amitriptyline and amoxapine), serotonin and norepinephrine reuptake inhibitors (SNRIs, such as desvenlafaxine and duloxetine), anti-convulsant drugs (such as carbamazepine, lamotrigine, and valproic acid), B12 vitamin methylcobalamin, pregabalin and gabapentin, may lead to adverse drug reactions. Potential adverse reactions associated with TCAs, SNRIs, pregabalin, and gabapentin include dizziness/light-headedness and drowsiness [35], which in older adults could lead to falls and related complications. TCAs, which are commonly prescribed, are also linked to an increased risk of heart diseases [36] and stroke.

Older adults with DPN often have complaints of reduced walking speed, impaired gait, and difficulties in stabilizing their body while walking on irregular surfaces [37]. The investigators of a multi-ethnic study have shown a consistent association of poor peripheral nerve (both sensory and motor) function and worse physical performance in older diabetic individuals [10]. It has been reported that DPN may compromise balance during daily activities, which further increases the risk of falling and associated injuries [37, 38]. Balance impairment due to DPN is also known to be associated with activity avoidance, institutionalization and mortality [39]. Thus, low physical function and impaired balance among older adults with DPN would have a detrimental effect on their mobility [11, 40], physical independence [10, 12–14, 37] and quality of life [11]. The TUG score used in the present study not only assesses the functionality of individuals with DPN but can also be used as a predictor of falls and frailty [41]. This study showed a significant improvement in the TUG score for both the DTLT (27%) and SLT (11%) groups, but there was a greater improvement in the DTLT group than in the SLT group. However, the difference in change between the groups was not statistically significant. While the greater improvement in the TUG score among the DTLT group may have arisen due to various contributing factors, such as reduction in pain and improvement in gait speed and physical performance, the significant improvement in the control group may be attributed to the health awareness counselling that they had undergone and their compliance with performing the recommended physical activities. Our finding corroborates the observations of Meneses et al. [42], where the authors noted a 20% improvement in the TUG score of patients aged between 50 and 75 years with knee osteoarthritis receiving low-level laser therapy.

DTLT reduced the levels of inflammatory markers in patients with DPN, which is important because a heightened inflammatory pathway is one of the contributory factors for pain and disability [43]. Our study revealed a statistically significant reduction in serum MCP-1 levels in the laser therapy group compared to the SLT group. The reduction in serum concentrations of IL-6 in the present study was also found to be greater in the DTLT group when compared to the SLT group. Similarly, the present study showed the reduction in serum concentrations of RANTES to be greater in the laser therapy group compared to the control group, although the decrease in RANTES concentration was statistically insignificant. Though there was a greater reduction in the serum concentrations of these inflammatory markers, the difference in the mean reduction was not statistically significant between the groups. Previous studies related to laser therapy have revealed that DTLT helps to decrease nociception through the reduction of inflammatory metabolites such as TNF-α, IL-6, prostaglandin E2 (PGE2), and cyclooxygenase-2 (COX-2) [17]. A study conducted in mice by Fukuda et al. [44] reported a cumulative effect in modulation of MCP-1 concentration following a minimum of three exposures to DTLT [44]. Kim et al. conducted a study to determine the effects of low intensity laser therapy on inflammatory osteoarthritis in the knee joint of rats and reported a significant decrease in serum IL-6 concentration in the laser therapy treatment group compared to that of the controls [45]. The possible mechanism responsible for the anti-inflammatory effects of deep tissue laser therapy in the present study could be due to the modulation of reactive oxygen species by the mitochondrial pathway, as reported previously. The signalling pathways (nitric oxide, cyclic AMP, calcium) [46] and the reduction or inactivation of NF-κB, as observed in a study on rats, might also have a role in the reduction of inflammation following DTLT [47]. The present study did not show any reduction in the serum concentration of TNF-*α* in the group that was treated with DTLT, and there was an increase in the serum concentrations of TNF-*α* in both groups. A possible hypothesis is that the dose of laser therapy is critical for reducing TNF-*α* release, as reported in previous studies [44]. Further, the chronic subtle inflammation that is associated with ageing [48], the small sample size and short duration of the present study may be other contributory factors for this finding.

The quality of life of pDPN patients is often poor due to impaired activities of daily living, and the nocturnal exacerbation of neuropathic pain [49, 50]. Pain can also isolate the older adults from social interactions and recreational activities, often leading to depression [8]. Our study showed a statistically significant improvement in the QOL of the group receiving DTLT, without any adverse events. An overall improvement was observed in the domains of physical functioning, physical, bodily pain, general health, vitality, social functioning, emotional, and mental health [30]. This finding is supported by the results of another pre-post designed study in which an improved neuropathic quality of life scale score was observed following the intervention [51], although the study included participants of various age groups. The improved QOL observed in the present study resulted from various factors, such as increased reduction in pain and inflammatory biomarkers and improved functionality, as assessed by the TUG scores in the DTLT group.

Our study revealed that patients’ quality of life was significantly improved in the DTLT group but not in the sham group. Pain levels were significantly lower in both groups and significantly lower in the DTLT group than in the sham group following the intervention. The Timed Up and Go test times were significantly shorter in both groups and shorter in the DTLT group than in the sham group (16%) following the intervention, although the difference was not significant. Serum levels of MCP-1 were significantly lowered in the DTLT group following the intervention, but not in the sham group. Serum levels of IL-6 were significantly lower in both groups following the intervention, and they were lower in the DTLT group than in the sham group, although the difference was not significant. Serum levels of TNF-***α*** and RANTES were not significantly changed in either group. Whether the participants’ responses to sham laser therapy were due to the placebo effect or to the institution’s (AIIMS) standard-of-care treatments cannot be ascertained from this study design. However, in principle, AIIMS, being a premier institute, has a well-established standard-of-care treatment that is safe and effective in its intent. Regardless, our study showed that DTLT did have a significant effect on the study’s primary outcome measures – pain and QOL.

### Strength of the study

To the best of our knowledge, this is the first randomized, double-masked, sham-controlled, interventional study of deep tissue laser therapy as an intervention for pain management, systemic inflammation, overall functionality and quality of life in older adults with pDPN.

### Limitation of the study

There are a few limitations of this study. 1) Our study lacks data on the duration of DPN. 2) There is a variable disease duration among study participants. 3) There is a lack of statistical power for some comparisons. Finally, this is a pilot study that requires validation in a multicentre trial with a larger cohort and a longer follow-up.

## Conclusion

Deep tissue laser therapy significantly reduced pain and improved the overall quality of life of older adults with painful diabetic peripheral neuropathy. Given the non-invasive nature of the intervention, its high compliance rate, and its safety profile (i.e., no known adverse or side effects), deep tissue laser therapy should be considered a safe, nonpharmacological addition to the standard of care for the management of pain in older adults with painful diabetic peripheral neuropathy. To further elucidate these findings, the authors recommend more randomized control trials in multiple settings, involving a larger cohort, and with a longer follow-up period.

## Data Availability

The datasets used and/or analysed during the current study are available from the corresponding author on reasonable request.

## Abbreviations

AIIMS: All India Institute of Medical Sciences
ATP: Adenosine Tri Phosphate
COX-2: Cyclooxygenase-2
CTRI: Clinical Trial Registry-India
DPN: Diabetic peripheral neuropathy
DTLT: Deep Tissue Laser Therapy
ELISA: Enzyme Linked Immunosorbent Assay
IEC: Institute Ethics Committee
IL-6: Interleukin-6
MCP-1: Monocyte Chemoattractant Protein-1
NCD: Non-Communicable Disease
NPS: Neuropathic Pain Scale
PDQ: Pain Disability Questionnaire
PGE2: Prostaglandin E-2
QOL: Quality of Life
QVAS: Quadruple Visual Analog Pain Scale
RANTES: Regulated on Activation, Normally T-Cell Expressed And Secreted
SD: Standard Deviation
SF-36: Short Form-36
SNRI: Serotonin and Nor epinephrine Reuptake Inhibitors
TCA: Tricyclic Antidepressants
TNF: Tumour necrosis factor
TUG: Time Up and Go test
